# Reassessment of Recycling Rates of Platinum Group Metals (PGMs) and Their Impact on Sustainability Models

**DOI:** 10.1021/acsomega.5c11916

**Published:** 2026-05-27

**Authors:** Margery Ryan, Antonio Zanotti Gerosa, Rupen Raithatha, Stewart Brown, Alison Cowley, Mikio Fujita, Nicolas Girardot, Athena Wang

**Affiliations:** † Johnson Matthey, Platinum Group Metal Services, Gate 2 Orchard Road, Royston SG8 5HE, U.K.; ‡ Johnson Matthey, Life Science Technologies, Platinum Group Metal Services, 28 Cambridge Science Park, Cambridge CB4 0FP, U.K.; § Johnson Matthey, Dai-ichi Life Hibiya First 12F, 1-13-2 Yurakucho, Chiyoda-ku, Tokyo 100-0006, Japan; ∥ Johnson Matthey, 588 Dong Xing Road, Song Jiang Industrial Zone, Shanghai 201613, China

## Abstract

A reliable estimate of the impact of recycling is crucial for a fact-based assessment of the current and future sustainability of platinum group metal (PGM)-based catalysts. Recycling rates in the literature are usually calculated based on published market data, notably from Johnson Matthey, but as these reported data exclude “closed-loop” recycling, this method leads to a significant underestimation of recycling volumes. Harnessing Johnson Matthey’s primary PGM market research and its proprietary suite of supply/demand/recycling models, we publish here an estimate of closed-loop recycling volumes. We find that closed-loop recycling volumes are much larger than open-loop recycling (which is reported in market data as secondary PGM supply) and that it is likely that between 50 and 60% of the metal used on new products is now sourced from recycling (globally on average). The significant reduction in global warming potential (GWP) of recycled (secondary) PGMs compared to newly mined (primary) metal means that an estimate of the proportion of recycled metal in a catalytic process or PGM-based product must be properly factored into LCA models. Our data and scenarios suggest that the GWP impact of PGMs can be reduced by 1 order of magnitude when a recycling “closed loop” is put in place within a process, underscoring the sustainability benefit of implementing routine recycling wherever practicable and ensuring that the recovered metal is retained.

## Graphical abstract

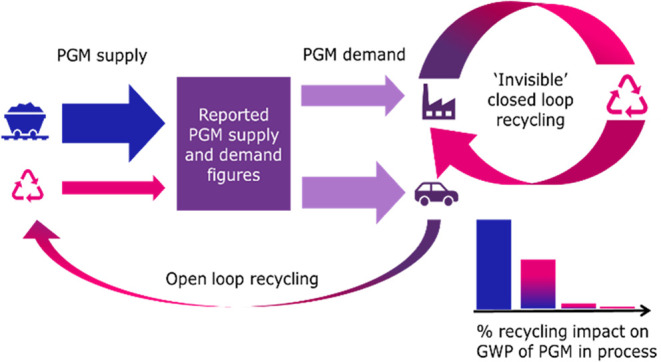

## 1 Introduction

Widely used as catalysts and as durable/high-temperature materials, platinum group metals (PGMs) have a critical role in sustaining modern life, enabling or enhancing key technologies within a wide range of human activities, from agriculture to transportation, healthcare to digital technologies.^1^ In particular, the sustainability of PGM-based catalysts in the production of fine chemicals, where they are critical to the synthesis of most active pharmaceutical intermediates (APIs), is the subject of intense debate. Different strategies are pursued, from a drive for replacement with earth-abundant metal (EAM)-based catalysts^2^ to attempts to implement an array of technologies for optimization, catalyst, and metal recovery.^3^ Any decision on the impact of PGM-based catalysts heavily relies on measuring their environmental footprint and understanding their contribution to the catalytic step that they enable.^4^

In this article, we provide a novel, reasoned assessment of the contribution of recycling to the current and future availability of PGMs and of the impact that recycling has on environmental impact models involving PGMs. In doing this, we build on the privileged know-how gained by Johnson Matthey, a company that, since its foundation in 1817, has been involved in trading, refining, fabrication, research, and market development of PGMs and PGM-based technologies. Johnson Matthey is also the world’s largest recycler of PGMs by volume.

PGMs have been in widespread industrial application for many decades, and supply chains are well-established. Similarly, PGM recycling networks are well-developed and mature. The early introduction of recycling was driven by the high value of these metals and facilitated by the fact that, unlike other materials such as plastics, recycling does not downgrade the properties of the PGMs.

A fact-based assessment of the current and future sustainability of PGM-based catalysts requires two critical pieces of intelligence: (a) a comprehensive life cycle analysis (LCA) of PGMs that distinguishes the impact of mined metal (primary) vs recycled metal (secondary); (b) a reliable estimation of the contribution of recycled metal to overall demand.

As per point (a), the International Platinum Group Metals Association (IPA) publishes updated numbers for the environmental impact of PGMs, distinguishing between the impact of primary metal (from mining) and secondary metal (from recycling).^5^ The most recent data set is for production year 2022; it comes from the contribution of the members of the IPA and covers over 95% of global primary and approximately 60% of global secondary (recycling) production. Purely focusing on the global warming potential (GWP, or carbon footprint, expressed in kg of CO_2_ per kg of metal), it is evident that the impact of recycled PGMs (specifically platinum, palladium, and rhodium, for which the IPA has published data on the secondary metal footprint) is a small fraction of the footprint of primary metals (Figure 1). A similar ratio can be assumed for iridium and ruthenium, which are largely mined as minor byproducts of platinum and are processed in the same facilities as the other three PGMs.^6^

**Figure 1:**
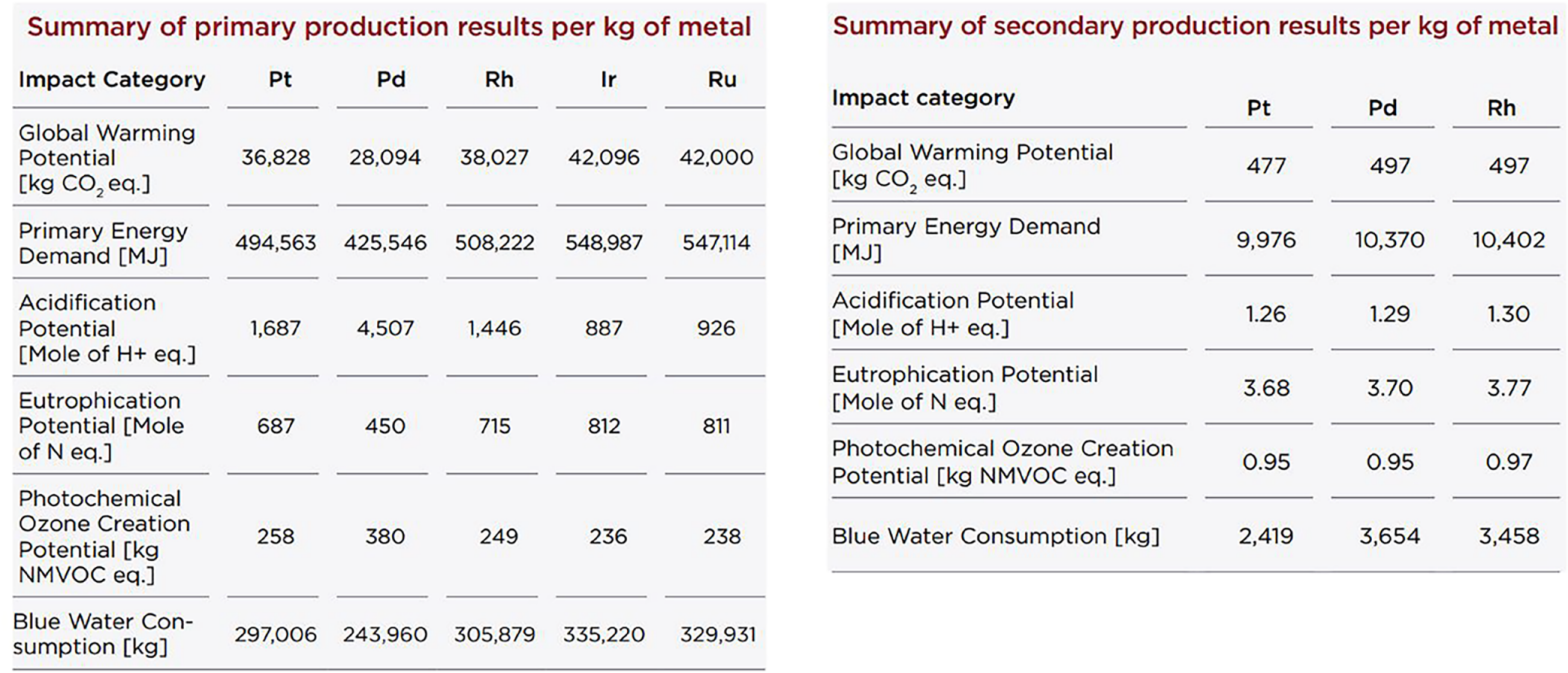
LCA results for primary and secondary production per kg of each metal. (Reprinted in part with permission from IPA Study “LCA on the global production of Platinum Group Metals, Platinum, Palladium, Rhodium, Iridium, and Ruthenium” for reference year 2022 and performed by Sphera. Copyright 2025, IPA.).

There is widespread interest in estimating the environmental footprint of PGMs but, given the significant difference in GWP between primary and secondary metal, previous attempts to estimate the carbon footprint of PGMs have heavily depended on assumptions made on the contribution of metal recycling.^7,8^ Different recycling percentages have been reported in the literature, often referring to sets of data that are limited to certain markets and geographies (e.g., palladium recycling from the automotive sector).^9–12^ In many cases, reported market data from Johnson Matthey^1^ or other market analysts are quoted to derive global recycling figures. This consistently leads to recycling proportions of around 20–30% being calculated for the PGMs, and/or statements that recycling from the automotive sector constitutes the bulk of total PGM recycling.

However, reported market data do not include figures for routine recycling and reuse of metal within the so-called closed loop, which is a feature of many PGM applications; the reason for this is that closed-loop reuse of metal retains metal ownership and does not change the balance of supply and demand in the PGM market. To provide a better estimate of total PGM recycling volumes, we report here a re-evaluation of total PGM recycling occurring yearly in the world based on information collected by the Johnson Matthey PGM Market Research group, including, for the first time, an estimated figure for closed-loop recycling.

Metal that is recovered in the closed loop does not need to be repurchased, so there is a substantial economic benefit to the applications that practice such recycling. Note, however, that no significant difference in GWP between metal recovered via open-loop recycling versus closed-loop recycling has been determined: the figures for secondary metal in Figure 1 can be applied to both recycling loops. The benefit of a closed loop does not lie in a difference in GWP per kilogram of metal. Rather, closed loops are known to improve recycling efficiency by increasing rates of collection of end-of-life material. Furthermore, they ensure that the original purchaser of the metal receives the full benefit of its recycling (rather than the recycled metal being placed on the market for purchase by other users). Therefore, implementing an effective closed loop should allow for a higher ratio of recycled metal to primary metal within an application than would otherwise be the case, lowering the overall GWP of the process.

It should be kept in mind that a comparison of recycling volumes to demand today gives insight into how much recycled metal is being used on new products (globally, on average), but it does not elucidate end-of-life recycling rates. Given significant growth in PGM consumption over the last few decades, and the long lifetime of many PGM products in use, recycling volumes today are reflective of previous years’ levels of PGM consumption, and lifetimes differ by application and by geography. We caution that a determination of end-of-life recycling rates requires a different approach from that taken in this paper and must be application- and possibly region-specific.

## 2 Methodology and Results

The Johnson Matthey PGM Market Research group is an international team of analysts that conducts primary (i.e., first-hand) research into PGM supply, demand, and recycling. It benefits from Johnson Matthey’s position in global supply chains, which allows for privileged insight into PGM use, but the team also conducts field research and maintains a network of industry contacts and information sources that is independent of Johnson Matthey’s commercial activities. It has been conducting PGM market research for over 50 years and has maintained a continuous supply/demand data set since the 1980s.^1^

The group uses a range of models to track and forecast trends in PGM supplies and applications globally for strategic purposes. These models are constructed to be bespoke for each demand area or supply source and, as far as possible, are constructed “bottom up,” rather than functioning “top down.” For example, in the case of PGM demand for automotive emissions catalysts, models are built up by individual vehicle models and by applicable catalyst loading (depending on the emissions regulation regime that pertains in the region where the vehicle will be first sold). These demand estimates then feed into recycling models: with knowledge of the age profile of vehicles at the end of life (the scrappage curve) and typical losses, it is possible to calculate how much metal is likely to be available for recycling from autoscrap each year.

To take a different example: in the case of application of a PGM catalyst to make a certain bulk chemical, the team will attempt to maintain a full list of all relevant plants that are in operation or under construction globally, the capacity of each plant, the catalyst requirement per unit of capacity, and the PGM loading on the catalyst. These data are sufficient to calculate the quantity of PGM installed in each plant and the total required for new capacity each year. Given the regular renewal of process catalyst charges, the models must allow for recycling, so further parameters such as catalyst lifetime, in situ losses, and any processing losses are built into the models to calculate PGM recycling volumes, as well as the demand for additional PGM to “top-up” losses. The recycling loop is an intrinsic aspect of forecasting PGM demand across many industrial applications. As such, the estimates for PGM recycling volumes provided by Johnson Matthey have largely been compiled “bottom up,” albeit top-down checks (for example, against estimated PGM refining capacities, which are not public knowledge) are applied as far as possible.

PGM recycling in most large industrial applications is routine. As the examples above suggest, two distinct PGM recycling pathways or mechanisms exist, one of which returns refined metal to general use (e.g., automotive catalysts), and the other allows for reuse of recovered metal by the owner or within the same application (e.g., industrial catalysts). These are typically termed the “open loop” and “closed loop,” respectively, and both contribute to PGM availability, but they are accounted for in different ways within reported market data.

Open-loop recycling supplies refined metal to the market in the same way that a primary mine would (albeit with a much lower GWP). It is therefore explicitly reported as “secondary supply,” for example, in the market data published by Johnson Matthey in May each year in its PGM Market Report.^13^ Meanwhile, by keeping metal in use rather than going to landfill, closed-loop recycling acts to reduce demand and thereby the need for additional supply to the market. PGM net demand is reported after subtracting any closed-loop reuse, and, as it is not counted toward secondary supply, closed-loop recycling is thus essentially invisible in reported market data. Nonetheless, the closed loop is a significant feature in PGM market dynamics as most PGM recycling in industrial applications, and virtually all recycling of ruthenium and iridium, takes place within a closed-loop pathway.

Open-loop recycling typically occurs with PGMs used in consumer markets, with the main source being the automotive industry, where palladium, platinum, and rhodium are recovered from scrapped catalytic converters. Smaller amounts of platinum and palladium are also recycled in an open loop from electronics scrap and old jewelry. For consumer products, the original purchaser of the PGM is typically the original equipment manufacturer (OEM), and any PGM contained within the product is sold with it. When the product is scrapped at the end of its life, the scrap is typically purchased by specialist collectors and recycled to recover any valuable products that can then be sold for profit. Due to the dispersed nature of end-of-life consumer products and the fact that the product owner often does not have any specific incentive to ensure the PGM is recovered, collection rates (and thus recycling rates) in the open loop tend to be less than optimal.

Closed-loop recycling typically occurs within industrial applications such as chemicals, fuels, and pharmaceutical production and glassmaking, and also applies to production scrap (for example, sputtering targets used in the production of hard disk media for data storage are routinely recycled once spent). Here, the original metal purchaser may be the OEM (for example, the catalyst manufacturer or fabricator of the PGM part) or the owner of the industrial facility in which the catalyst or part will be installed; the point is that the metal will likely stay in place within the ownership of that facility for its full life cycle. At the end of its life, the facility operator has an interest in sending the spent catalyst or equipment for recycling. Any recovered metal will be offset against the metal required to fabricate the product that replaces the spent catalyst charge or equipment. It thus reduces the amount of new PGM that must be bought to maintain the installed capacity, although small losses through the life cycle and recycling process mean that some purchase of “top-up” metal is usual.

To estimate the proportion of recycled (secondary) versus newly mined (primary) metal used annually on newly fabricated PGM products requires examination of the interplay between demand and supply. The following definitions are used:

- **market supply** = annual sales of mined PGMs + open-loop recycling.
- **market demand** = annual demand for PGM (net of the closed loop), which may be different from the market supply, leading to surpluses or deficits in individual years.
- **estimated gross demand** = annual market demand + closed-loop reuse (note that no deficit or surplus is considered in the closed loop, i.e., it is assumed that closed-loop recycling and closed-loop reuse are identical in a particular year).
- **Total recycling** = annual closed-loop recycling + open-loop recycling.

The calendar year 2024 was chosen as the reference year for this determination.^13^

### 2.1 Contribution of Secondary Supply (Open-Loop Recycling) to Total Supply

Johnson Matthey reported PGM supplies for 2024, as shown in Table 1. Figures have been converted from troy ounces and rounded to the nearest metric ton.^13^

**Table 1:** PGM Supplies in 2024 as Reported in May 2025^a^

	platinum (mt)	palladium (mt)	rhodium (mt)	ruthenium (mt)	iridium (mt)	combined PGM (mt)
primary supply (from mining)	178	207	22	35	7	449
secondary supply (from open-loop recycling)	43	91	9	ND^b^	ND^b^	143
total market supply	221	298	31	35	7	592

a Adapted with permission from Ref 13, Copyright 2025, Johnson Matthey.

b Ruthenium and iridium lack consistent sources of secondary supply, and they are typically considered to be recycled in a closed loop; therefore, Johnson Matthey has, to date, not published a secondary supply figure for these two metals.

It can be seen that in 2024, recycled metal contributed an estimated 19.5% of total platinum supplies, 30% of total palladium supplies, 29% of total rhodium supplies, and 24% of total supplies on a combined PGM basis. These are typical proportions for recent years and are broadly in agreement with published statements on PGM recycling, which often reference Johnson Matthey PGM reports.^9–12^

### 2.2 Market (Net) Demand

Johnson Matthey reported PGM demand for 2024 as shown in Table 2 (converted to metric tons), along with the market balance (supply less demand).^13^

**Table 2:** PGM Demand in 2024 as Reported in May 2025^a^

	platinum (mt)	palladium (mt)	rhodium (mt)	ruthenium (mt)	iridium (mt)	combined PGM (mt)
reported net demand^b^	228	307	33	37	7	613
market balance^c^	–7	–9	–2	–2	0	–21

a Adapted with Permission from Ref 13, Copyright 2025, Johnson Matthey.

b Excludes investment demand.

c It is typical in the PGM market for supply and demand not to balance in a particular year, and either a surplus (positive market balance) or a deficit (negative market balance) is reported. Surplus supplies increase market stocks, and these are drawn on to fill deficits in subsequent years. Physical metals held by investors also function as a form of stock.

### 2.3 Estimated Closed-Loop Circulation of PGMs

Johnson Matthey does not routinely report closed-loop data. In early 2025, for the first time, in a white paper, Johnson Matthey published estimates for closed-loop recycling volumes in 2024, based on data collated in December 2024 (Table 3).^14^

**Table 3:** Estimated PGM Closed-Loop Recycling Volumes in 2024^a^

	platinum (mt)	palladium (mt)	rhodium (mt)	ruthenium (mt)	iridium (mt)	combined PGM (mt)
estimated closed-loop recycling	294	87	27	42	12	462

a Adapted with Permission from Ref 14, Copyright 2025, Johnson Matthey.

Estimating closed-loop recycling is subject to greater uncertainty than open-loop recycling, since sources are far more diverse and volumes arising from equipment changeouts or catalyst replacements tend to be “lumpier,” with the timing of this “lumpiness” being opaque. In Johnson Matthey’s modeling, catalyst/equipment changeouts tend to be averaged over lifetimes to smooth out this lumpiness. Plant or capacity closures may contribute additional lumpiness in some years, but in a much less predictable way. Although metal released from closures may be sold back to the market by the owner, thereby constituting open-loop recycling, this is not a consistent source and is therefore not reported as secondary supply by Johnson Matthey. Instead, capacity closures or inventory releases are counted as “negative demand” and counted toward the market demand number. This approach ensures no double-counting of metal (since “negative demand” has the same effect on the market balance as “positive supply”). However, here we are concerned with total recycling volumes and, in order to account for any recycling of released inventory in 2024, it is included within the closed-loop estimates.

### 2.4 Estimated Gross Demand

To derive a figure for gross demand, the above estimate for closed-loop recycling is taken as a proxy for the amount of metal reused in the closed loop. In reality, small losses through the recycling and refining processes mean that the amount of metal input to the closed loop will be somewhat less than the output. However, the uncertainty associated with the closed-loop estimates (discussed below) is comfortably greater than the lost volume, which can therefore be ignored for the purposes of this calculation.

Taking the closed-loop estimate above and adding Johnson Matthey’s reported net demand in 2024 provides the gross demand estimate shown in Table 4.

**Table 4:** Estimated Gross PGM Demand in 2024^a^

	platinum (mt)	palladium (mt)	rhodium (mt)	ruthenium (mt)	iridium (mt)	combined PGM (mt)
reported net demand^b^	228	307	33	37	7	613
estimated closed-loop reuse	294	87	27	42	12	462
estimated gross demand	522	394	60	79	19	1075

a Adapted with permission from Ref 14, Copyright 2025, Johnson Matthey.

b Excludes investment demand.

### 2.5 Total Contribution of Recycling to Gross Consumption of PGMs

Combining the data (Table 5) allows for an estimate of the contribution of recycling to the overall use of PGMs in 2024.

**Table 5:** Estimated Contribution of Recycling to Overall Use of PGMs in 2024^a^

	platinum (mt)	palladium (mt)	rhodium (mt)	ruthenium (mt)	iridium (mt)	combined PGM (mt)
estimated gross demand	522	394	60	79	19	1075
estimated closed-loop recycling	294	87	27	42	12	462
secondary supply (open-loop recycling)	43	91	9	ND	ND	143
total recycling	337	178	36	42	12	605
proportion of gross demand met by recycling (%)	65	45	60	53	63	56

a Adapted with permission from Ref 14, Copyright 2025, Johnson Matthey.

## 3 Discussion

The proportion of recycling varies by metal, being the highest at an estimated 65% for platinum and the lowest at 45% for palladium. This reflects variations in the applications of the different metals. For example, platinum, along with rhodium, is used in significant quantities in coatings and fabrications for glassmaking (specifically, fiberglass, display panel glass, and specialty glass), which are associated with high recycling rates and large closed loops. Palladium has minimal application in glassmaking but sees widespread use in electronics and dental applications, in which end-of-life recycling rates tend to be relatively low. The major use of palladium is in the automotive market, and the less-than-optimal extent of recycling in the automotive sector has been analyzed in several sources.^10,14^

Across the five PGMs, the estimated proportion is around 56%. It is worth considering that any presumed uncertainties of roughly ±25% in the estimates of closed-loop size (and the consequent uncertainty in the estimates of gross demand) would only result in an uncertainty of ±5% on this figure. Recycling is thus a substantial contributor to the amount of PGM used in the fabrication of new catalysts, parts, and equipment, safely estimated between 50 and 60%.

### 3.1 Recycling Scenarios

Based on the data presented in Figure 1 and Table 5, any study aimed at establishing the impact of PGMs on the GWP of specific industrial processes may choose to consider four different scenarios. Figure 2 illustrates some options for platinum and for palladium.

- **100% Primary Metal**
- **65% (Pt) or 45% (Pd) secondary metal**, i.e., assuming metal is sourced in line with the average obtained from the results above.
- **100% Secondary Metal**
- **98% secondary metal**, i.e., for a theoretical case, based on industry best practice, where highly efficient and nearly ideal closed-loop recycling is practiced and 2% top-up metal is purchased from primary sources.

**Figure 2:**
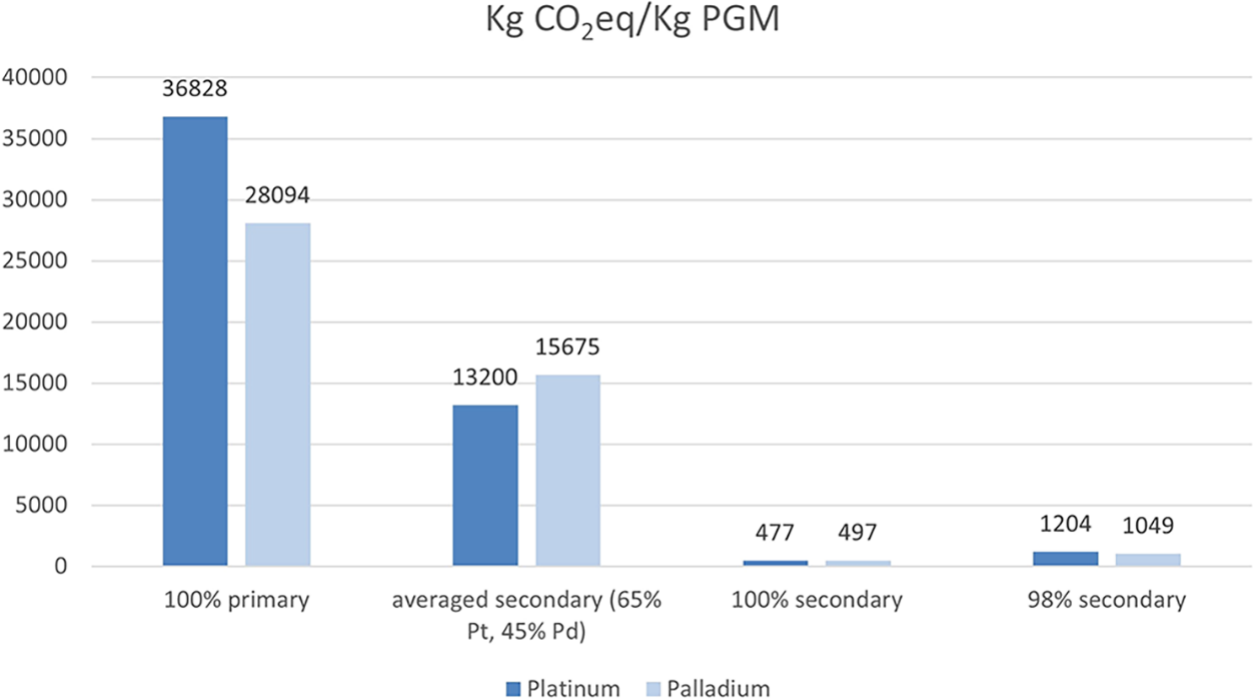
Impact of recycling on global warming potential per kilogram of platinum or palladium in four sourcing scenarios.

The first scenario (100% primary) would apply, for example, during the first life cycle of a product for which metal has been purchased directly from a mining company. In the case of a PGM catalyst purchased from a catalyst supplier (e.g., a 5%Pd/C), this scenario is unrealistic as, in practice, the metal utilized by the catalyst manufacturer is likely to be a mixture of primary and secondary metal.

The second scenario (averaged secondary) does not consider any specific sourcing arrangement or recycling embedded in a specific process but allows for the fact that, globally, on average and across all applications, about 65% (platinum)/45% (palladium) comes from secondary sources. In fine chemical applications, this scenario is, in the absence of any established metal recycling data for the process, a reasonable assumption to assess the GWP of the metal utilized in a catalytic process. The GWP of the metal, often, especially for heterogeneous catalysts, can approximate the GWP of the entire catalyst. While the ratio between primary and secondary sources will vary among different catalyst suppliers, the numbers estimated in this work aim at providing a global average, independent of geography and specific markets.

The third scenario (100% secondary) may occur where a customer requires metal to be certified as 100% recycled, which can be done for a particular batch of metal on a mass-balance basis, i.e., by matching the metal sale to purchase or sourcing from a known secondary input, and/or by retaining the metal in a closed loop. In practice, of course, there is a limit to the amount of secondary metal available in the market, so there is an upper limit to the number of customers that can receive recycled metal; the remaining demand must be satisfied with primary metal.

The fourth scenario (98% secondary) reflects a theoretical case of metal reuse within a specific closed loop but allows for some real-world processing losses (assumed to be 2% end-to-end) and assumes the customer purchases the top-up metal exclusively from a primary source. In the case of an industrial process where the metal is used as a catalyst, additional losses could happen during reaction, workup, collection of spent catalyst, etc., but the required top up from primary metal is likely to remain a fraction of the total metal employed in each life cycle (and there is also the option of purchasing secondary metal for top up, or a combination of primary and secondary). In the fine chemicals space, this scenario would most closely describe a situation where the catalyst is refined in a closed loop, highlighting that, whenever possible, recovery and refining of the metal component of a catalyst are critical for the sustainability of the overall process.

Overall, the significantly lower GWP of the scenarios considering metal recycling underscores the benefit of implementing a closed loop wherever practicable.

### 3.2 Future Trends

A relevant question is to what extent the proportion of recycled metal in the market can be increased, and whether a case of 100% secondary metal for all PGM uses is ever likely to be achieved. As noted above, there are small losses inevitably incurred in any real-world processing and therefore recycling rates in certain applications may approach 100% but will not reach it. The more significant factors in end-of-life recycling rates, however, tend to be either losses during use or losses due to inefficient collection of end-of-life material that is otherwise a candidate for recycling. Addressing those losses could improve recycling rates and increase the volumes of recycled metal available for use. For example, PGM in catalytic converters on the world’s vehicle fleet represent a very substantial “urban mine” of PGMs which can be exploited for decades to come via recycling and there is scope for improvement in recycling rates.^10,14,15^

Even with minimized collection losses, however, application-related losses will persist and make it highly unlikely that 100% reliance on recycling can ever be achieved, even in the absence of further growth in PGM demand. Some application-related losses are in fact considered to be acceptable. Given their superior durability, PGMs are applied in many settings where other materials would not perform adequately. However, harsh conditions may still lead to PGM losses in situ: for example, certain electrochemical process applications tend to see some metal lost to the liquor from the electrode coatings over time. In certain other instances, PGMs are designed to be used in a way that means they are unrecoverable, the main example here being platinum-catalyzed hydrosilylation with Karstedt’s catalyst: the platinum catalyst is entrained in the silicone and thus becomes dispersed at ultralow concentrations in the product of the process. Another relevant example is platinum-based anticancer therapeutics. Together, these two applications account for more than 10 tonnes of platinum consumption annually.

As such, there is an intrinsic limit to how far recycling can go to improve the LCA of PGMs. Primary PGMs will not be eliminated in the market and are particularly necessary to support any further growth in PGM consumption in new technology areas such as hydrogen production. Two factors, however, will work toward a downward trajectory of the carbon footprint of PGMs:

- The opportunity for increasing the efficiency of recycling (discussed above) and
- The progressive decarbonization of the energy supply that is utilized for mining and processing ores and recycling metals.

On the latter point, it is relevant to note that the GWP of primary PGMs is expected to decrease significantly by 2030 (by between 30 and 60%, vs the 2022 production year) due to the decarbonization of electricity generation in South Africa, the world’s biggest supplier of PGMs.^5^ Further improvement in the longer term is likely, as most of the major South Africa PGM mining companies have announced carbon emission reduction targets beyond 2030 and are also investing directly in renewable power supply.^16–19^

## 4 Conclusions

The publication of estimates of closed-loop recycling of PGMs, based on Johnson Matthey market models, allows a more realistic view of the total contribution of recycling toward the gross demand of PGMs every year. Combined with GWP numbers that clearly distinguish between the footprints of primary and secondary metals, the data presented here provide the basis for improved LCA models for industrial applications of PGMs. The carbon footprint contribution of PGMs, in fact, shows very substantial variations based on which recycling scenario is considered (from single-use metal to full closed-loop recycling) and which origin of metal is considered (mined, recycled, or any intermediate average). In the specific case of the use of PGMs as industrial catalysts, the scenarios offered clearly highlight the positive impact of establishing closed-loop recycling. In addition to identifying the correct scenario for any application being modeled prior to plugging in GWP figures for PGMs, we recommend that both GWP and recycling data are reviewed regularly, as they are likely to change following trends of increased recycling and power decarbonization.
